# Prevalence of Extended Spectrum Betalactamase (ESBL) and Metallobetalactamase (MBL) Producing* Pseudomonas aeruginosa* and* Acinetobacter baumannii* Isolated from Various Clinical Samples

**DOI:** 10.1155/2018/6845985

**Published:** 2018-10-24

**Authors:** Amandeep Kaur, Satnam Singh

**Affiliations:** ^1^Department of Microbiology, Adesh Institute of Medical Sciences and Research, Bathinda, India; ^2^Department of Pharmacology, Adesh Institute of Medical Sciences and Research, Bathinda, India

## Abstract

This study was conducted with an objective to find the prevalence of extended spectrum betalactamase (ESBL) and metallobetalactamase (MBL) in* P. aeruginosa* and* A. baumannii* isolates obtained from various clinical samples. It was conducted in the Department of Microbiology, Adesh Institute of Medical Sciences and Research, Bathinda, over a period of two years from July 2014 to June 2016. Clinical specimens including urine, pus, blood, high vaginal swabs, respiratory samples, and various body fluids were processed and* P. aeruginosa* and* A. baumannii* isolates were identified by standard protocols. Antibiotic sensitivity testing for all isolates was done using Kirby-Bauer disc diffusion method. Disc potentiation test was performed to check ESBL and MBL production in these bacteria. Maximum ESBL positive isolates of* P. aeruginosa *were observed among pus samples and maximum MBL positive isolates were detected in tracheal aspirates.* A. baumannii *showed maximum positivity for ESBL and MBL production in endotracheal secretions. This study gives an alarming sign towards high prevalence of cephalosporin and carbapenem resistance due to production of extended spectrum betalactamases and metallobetalactamases, respectively. Early detection, stringent antibiotic policies, and compliance towards infection control practices are the best defenses against these organisms.

## 1. Introduction

Resistance to antimicrobials is common and has increased over the years among* Pseudomonas aeruginosa *(*P. aeruginosa*) and* Acinetobacter baumannii* (*A. baumannii*) as a number of strains are now resistant nearly to all commonly used antibiotics. Multidrug resistance among these organisms makes the treatment of infections caused by them difficult and expensive. These bacteria are known to produce extended spectrum betalactamases (ESBLs) and metallobetalactamases (MBLs) [1]. The large scale use of the third-generation cephalosporins like cefotaxime, ceftriaxone, and ceftazidime has led to the evolution of newer betalactamases such as the ESBLs. ESBLs are plasmid mediated enzymes that hydrolyze the oxyimino *β* lactams and the monobactams (aztreonam) but have no effect on the cephamycins (cefoxitin, cefotetan) and the carbapenems (imipenem). Being plasmid mediated, they can be easily transferred from one organism to another [2]. Due to frequent resistance to aminoglycosides, fluoroquinolones, ureidopenicillins, and third-generation cephalosporins, carbapenems are important agents for managing such infections. Carbapenem resistance in* P. aeruginosa *and* A. baumannii *is attributed to various causes such as reduced expression of outer membrane proteins and carbapenamases [3]. MBL producing* P. aeruginosa* and* A. baumannii* have become a growing therapeutic concern worldwide. The rapid detection of MBL positive isolates is necessary to control infection and to prevent their dissemination [4]. The aim of this study was to determine the prevalence of ESBL and MBL production among strains of* P. aeruginosa* and* A. baumannii *obtained from various clinical samples.

## 2. Material and Methods

### 2.1. Study Design and Samples

The laboratory based prospective study was conducted in Department of Microbiology, Adesh Institute of Medical Sciences and Research (AIMSR), Adesh University, Bathinda, for a period for two years from July 2014 to June 2016 after getting approval from the Thesis Research Degree Committee and Ethical Committee of Adesh University. Written consent in the language known to the patient or their guardians was taken before including the patient in this study. Clinical history of the patient was taken regarding age, sex, date of admission, immune status, antibiotic treatment, date of discharge, etc. and recorded in the patient pro forma. A total of 2261 clinical samples were collected from patients admitted in ICU and various wards of the hospital depending upon the clinical diagnosis of respective patients. These included urine, pus, blood, ear swabs, high vaginal swabs, sputum, endotracheal secretions, tracheal aspirate, and various body fluids. All samples were collected as per standard microbiological guidelines [5].

### 2.2. Laboratory Methods

Various samples collected were inoculated onto Blood Agar (BA) and MacConkey Agar (MA) plates under strict aseptic conditions. Plates were incubated at 37°C for 24-48 hours under aerobic conditions. Provisional identification of* P. aeruginosa* and* A. baumannii* was done on the basis of Gram staining morphology and colony characteristics on Blood Agar and MacConkey agar media. A battery of biochemical tests were performed for confirmation of the isolates [6].

### 2.3. Antimicrobial Susceptibility Testing

Antimicrobial sensitivity testing of the isolates was determined on Mueller Hinton Agar by Kirby Bauer disc diffusion method [7]. The following antibiotic discs were tested: ceftazidime (30*μ*g), cefepime (30 *μ*g), piperacillin-tazobactam (100*μ*g/10 *μ*g), Imipenem (10 *μ*g), Meropenem (10 *μ*g), Gentamicin (10 *μ*g), Amikacin (30 *μ*g), Ampicillin-sulbactam (10 *μ*g/10 *μ*g), Cotrimoxazole (25 *μ*g), aztreonam (30 *μ*g), Ciprofloxacin (5 *μ*g), Norfloxacin (30 *μ*g) (for urinary isolates), Polymyxin B (300 units), and Colistin (10 *μ*g). Zone sizes obtained were measured and interpretation was made according to CLSI guidelines [8].* Pseudomonas aeruginosa* ATCC 27853 was used as the control organism for antibiotic sensitivity.

### 2.4. Phenotypic Detection of ESBL Positive Isolates

Isolates resistant to ceftazidime and/or cefepime were tested for ESBL production by disc potentiation test. A disc of ceftazidime (30*μ*g) and ceftazidime + clavulanic acid (30*μ*g/10 *μ*g) was placed 20 mm apart, centre to centre on Mueller Hinton agar plate, and was incubated overnight at 37°C. A zone difference greater than or equal to 5 mm around ceftazidime and ceftazidime + clavulanic acid was interpreted as ESBL positive isolate [9].

### 2.5. Phenotypic Detection of MBL Positive Isolates

Isolates resistant to imipenem and/or meropenem were tested for MBL production by disc potentiation test. A disc of imipenem (10*μ*g) and imipenem + EDTA (10*μ*g/750 *μ*g) was placed 20 mm apart, centre to centre on Mueller Hinton agar plate, and was incubated overnight at 37°C. A zone difference greater than or equal to 7 mm around imipenem and imipenem + EDTA disc was interpreted as MBL positive isolate [10].

**Figure 1:**
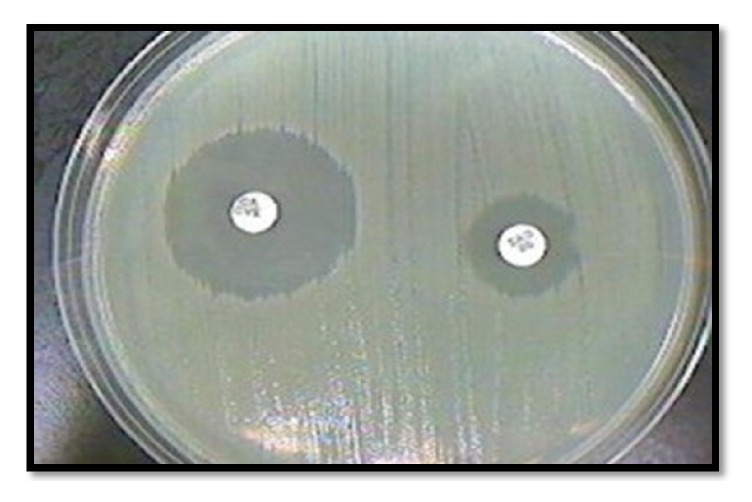
Disc potentiation test for detection of ESBL using ceftazidime (Right) and ceftazidime-clavulanic acid disc (left).

**Figure 2:**
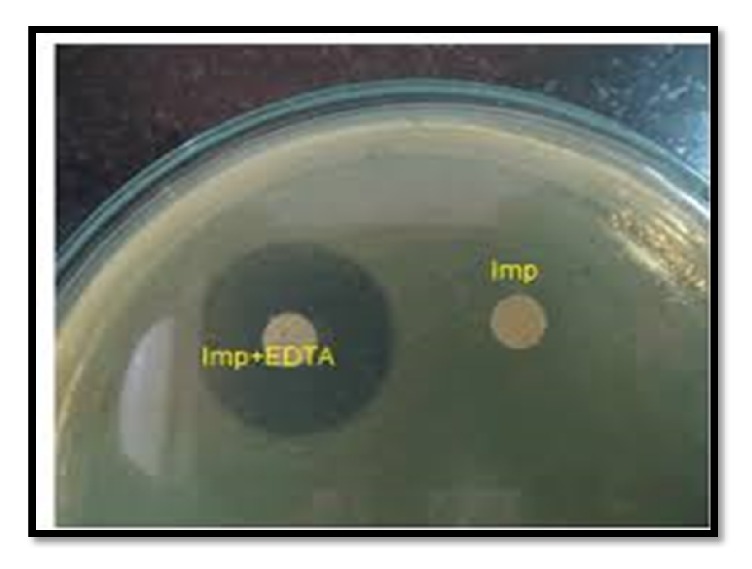
Disc potentiation test for detection of MBL using imipenem and imipenem-EDTA disc.

Disc potentiation test performed to check ESBL production and MBL production is shown in Figures 1 and 2.

### 2.6. Statistical Analysis

Statistical analysis was done by descriptive statistics using percentages and ratios methods and bar graphs were prepared in Microsoft Excel to represent antimicrobial susceptibility, ESBL, and MBL production.

## 3. Results

Out of total samples processed, 192* P. aeruginosa* and 116* A. baumannii* were isolated.

**Figure 3:**
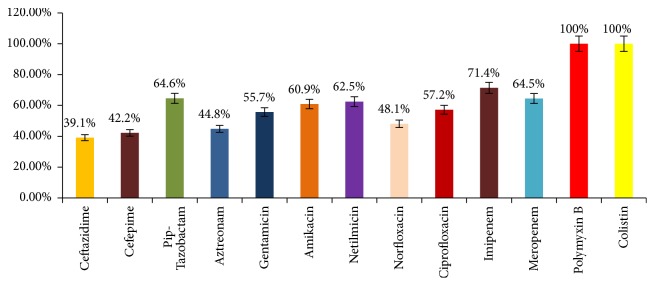
Antimicrobial susceptibility profile of* P. aeruginosa* (N=192).

*P. aeruginosa* isolates were 100% sensitive to polymyxin B and colistin, and 71.4% sensitivity was reported towards imipenem whereas intermediate sensitivity (64.5%) was recorded towards piperacillin-tazobactam and meropenem. Norfloxacin was tested only against urinary isolates and sensitivity was recorded as 48.4%. Much less sensitivity was reported towards cephalosporins: ceftazidime and cefepime, i.e., 39.1% and 42.2%, respectively. Antibiogram obtained for P.* aeruginosa* isolates is shown in Figure 3.

**Figure 4:**
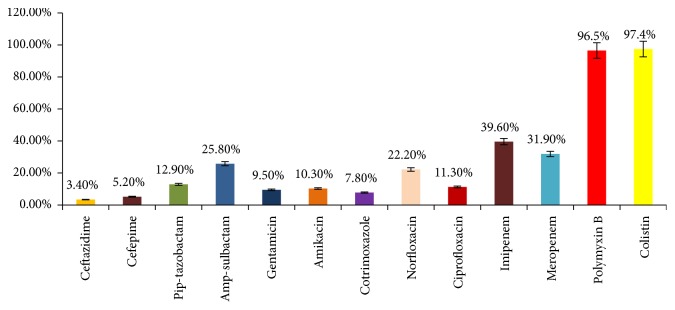
Antimicrobial susceptibility profile of* A. baumannii *(N=116).

*A. baumannii* isolates showed high level of resistance to most of the antibiotics tested. 96.6% strains of* A. baumannii* were found resistant towards ceftazidime and 94.8% strains were resistant towards cefepime. Resistance towards imipenem and meropenem was found to be 60.3% and 68.1%, respectively. However, 96.5% sensitivity was recorded for polymyxin B and 97.4% for colistin. Antibiogram obtained for* A. baumannii *is shown in Figure 4.

Antimicrobial susceptibility of* P. aeruginosa* and* A. baumannii *according to the various sampling sites is shown in Tables 1 and 2, respectively.

Out of 192 isolates of* P. aeruginosa*, ESBL production was seen in 34 (17.7%) isolates and MBL production was observed in 42 (21.8%) isolates as shown in Table 3. Out of 116 isolates of* A. baumannii*, ESBL production was seen in 32 (27.5%) isolates and MBL production was observed in 52 (44.8%) isolates as shown in Table 4.

## 4. Discussion 

In case of* P. aeruginosa,* maximum susceptibility to antibiotics was observed in case of pus, urine, high vaginal, and ear swabs whereas much less susceptibility was reported in case secretions/aspirates obtained from endotracheal and tracheostomy tubes.

Similarly,* A. baumannii *isolates obtained from ET secretions and tracheal aspirates showed very poor susceptibility to cephalosporins, aminoglycosides, fluoroquinolones, and even carbapenems. Isolates obtained from other sites were relatively sensitive as compared to respiratory sites.

Most of the patients admitted in ICU were put on mechanical ventilation due to severe head injuries and cerebrovascular accidents leading to prolonged antibiotic therapy. Longer stay of patients in ICUs, prolonged antibiotic treatment, and selective pressure in ICU environment could be the associative factors responsible for this variation as well as production of ESBLs and MBLs by this particular group of isolates.

In the present study, 17.7% isolates of* P. aeruginosa* were positive for production of ESBL production. The results are similar to study by Senthamarai et al. [11] who had reported ESBL production in* P. aeruginosa* as 22.2%. Goel et al. [12] and Rani et al. [13] have reported ESBL production in* P. aeruginosa* as 42.3% and 37.2%, respectively, which is slightly higher as compared to this study. Woodford et al. [14] and Lim et al. [15] have reported ESBL production in these bacteria to be very low, i.e., 3.7% and 4.2%, respectively. In this study, 21.8% isolates of* P. aeruginosa* were positive for production of MBL production. The results are similar to Upadhyay et al. [16] who had reported MBL production in* P. aeruginosa* as 20.8%. Sadhna et al. [10], Madhu et al. [17], and Behera et al. [18] have reported MBL production in* P. aeruginosa* as 41.0%, 61.5%, and 69.5%, respectively, which is higher as compared to this study. Aggarwal et al. [19] had reported MBL production to be 11.4% which is lesser as compared to this study. In the present study, 27.5% isolates of* A. baumannii *were positive for production of ESBL production. The results are exactly similar to Sinha et al. [20] who had also reported ESBL production in* A. baumannii *as 27.5%. Vahaboglu et al. [21] and Chaudhry and Payasi[22] have reported ESBL production in* A. baumannii* as 46.0% and 83.6%, respectively, which is higher as compared to this study. Bali et al. [23] had reported ESBL production to be 5.2% which is much lesser as compared to this study. In our study, 44.8% isolates of* A. baumannii *were positive for production of MBL production. The results are similar to Irfan et al. [24] who had also reported MBL production in* A. baumannii *as 49.0%. Hodiwala et al. [4] and Kabbaj et al. [25] have reported very high MBL production in* A. baumannii* as 96.6% and 74%, respectively. Gupta et al. [26] had reported MBL production to be 7.5% which is much lesser as compared to this study. These variations could be due to difference in the antibiotic usage and judicious selection of antibiotics in their hospital settings.

## 5. Conclusion

The present study shows that ESBL and MBL production in* P. aeruginosa* and* A. baumannii* is on the rise across the globe, thus making these infections difficult to treat. Early detection of ESBL and MBL production would be important for the reduction of mortality rate and spread of multidrug resistant organisms. The disc potentiation test is simple, easy to perform, and economical and can be done along with routine antibiotic sensitivity testing. Moreover, it is important to implement antibiotic restriction policies to avoid excessive and injudicious use of extended spectrum cephalosporins and carbapenems in every hospital.

## Figures and Tables

**Table 1:** Antimicrobial susceptibility of *P. aeruginosa *(N=192) in relation to various sampling sites.

**Name of Antibiotic**	**Pus** **(54)**	**Urine** **(54)**	**Blood** **(4)**	**Tracheal Aspirate** **(29)**	**Endotracheal secretions** **(20)**	**High vaginal swabs & Ear swabs** ** (17)**	**Various Body fluids** **(4)**	**Sputum** **(10)**
Ceftazidime	46.3%	42.6%	50.0%	13.8%	25.0%	58.8%	50.0%	40.0%
Cefepime	29.6%	59.3%	50.0%	20.7%	30.0%	70.6%	50.0%	50.0%
Piperacillin-Tazobactum	61.1%	81.5%	75.0%	41.4%	55.0%	70.6%	75.0%	60.0%
Aztreonam	55.6%	59.3%	50.0%	10.3%	20.0%	41.2%	50.0%	60.0%
Gentamicin	61.1%	68.5%	50.0%	20.7%	25.0%	82.4%	50.0%	80.0%
Amikacin	75.9%	70.4%	50.0%	20.7%	35.0%	82.4%	50.0%	70.0%
Netilmicin	70.4%	81.5%	50.0%	24.1%	40.0%	76.5%	75.0%	50.0%
Norfloxacin	NT	48.1%	NT	NT	NT	NT	NT	NT
Ciprofloxacin	70.4%	NT	75.0%	24.1%	40.0%	88.2%	50.0%	60.0%
Imipenem	79.6%	90.7%	75.0%	27.6%	45.0%	82.4%	100.0%	40.0%
Meropenem	79.6%	70.4%	50.0%	27.6%	30.0%	88.2%	100.0%	30.0%
Polymixin B	100.0%	100.0%	100.0%	100.0%	100.0%	100.0%	100.0%	100.0%
Colistin	100.0%	100.0%	100.0%	100.0%	100.0%	100.0%	100.0%	100.0%

**Table 2:** Antimicrobial susceptibility of *A. baumannii *(N= 116) in relation to various sampling sites.

**Name of Antibiotic**	**ET Secretions** **(34)**	**Tracheal Aspirate** ** (28)**	**Pus** ** (29)**	**Urine** ** (9)**	**Sputum** ** (7)**	**Blood** ** (6)**	**Various body fluids** ** (3)**
Ceftazidime	0.0%	0.0%	0.0%	22.2%	28.6%	0.0%	0.0%
Cefepime	0.0%	0.0%	3.4%	22.2%	28.6%	0.0%	33.3%
Piperacilllin- Tazobactum	0.0%	3.6%	13.8%	55.6%	28.6%	33.3%	33.3%
Ampicillin-Sulbactum	5.9%	17.9%	41.4%	66.7%	42.9%	33.3%	33.3%
Gentamicin	0.0%	0.0%	10.3%	44.4%	57.1%	0.0%	0.0%
Amikacin	0.0%	0.0%	10.3%	44.4%	42.9%	33.3%	0.0%
Cotrimoxazole	0.0%	0.0%	6.9%	55.6%	28.6%	0.0%	0.0%
Norfloxacin	NT	NT	NT	22.2%	NT	NT	NT
Ciprofloxacin	5.9%	14.3%	13.8%	NT	28.6%	16.7%	0.0%
Imipenem	20.6%	21.4%	75.9%	77.8%	42.9%	0.0%	33.3%
Meropenem	17.6%	21.4%	62.1%	22.2%	57.1%	0.0%	33.3%
Polymyxin B	91.2%	96.4%	100.0%	100.0%	100.0%	100.0%	100.0%
Colistin	94.1%	96.4%	100.0%	100.0%	100.0%	100.0%	100.0%

**Table 3:** Sample-wise distribution of ESBL and MBL isolates of *P. aeruginosa*.

No.of isolates	Urine	Ear swabs	Blood	Pus	ET secretions	Tracheal aspirate	Sputum
ESBL positive	3	1	1	9	8	8	4
(34)							

MBL positive	6	1	1	8	10	12	4
(42)							

**Table 4:** Sample-wise distribution of ESBL and MBL isolates of *A. baumannii*.

No.of isolates	Urine	Blood	Pus	ET secretions	Tracheal aspirate	Sputum
ESBL positive	4	3	3	12	8	2
(32)						

MBL positive	6	3	6	17	18	2
(52)						

## Data Availability

The data used to support the findings of this study are included within the article.

## References

[1] Memish Z. A., Shibl A. M., Kambal A. M., Ohaly Y. A., Ishaq A., Livermore D. M. (2012). Antimicrobial resistance among non-fermenting gram-negative bacteria in Saudi Arabia. *Journal of Antimicrobial Chemotherapy*.

[2] Okesola A. O., Oni A. A. (2012). Occurrence of Extended-Spectrum Beta-Lactamase-Producing Pseudomonas aeruginosa Strains in South-West Nigeria. *Research Journal of Medical Sciences*.

[3] Gladstone P., Rajendran P., Brahmadathan K. N. (2005). Incidence of carbapenem resistant nonfermenting gram negative bacilli from patients with respiratory infections in the intensive care units. *Indian Journal of Medical Microbiology*.

[4] Hodiwala A., Dhoke R., Urhekar A. D. (2013). Incidence of metallo-beta-lactamase producing Pseudomonas, Acinetobacter and enterobacterial isolates in hospitalised patients. *International Journal of Pharmacy and Biological Sciences*.

[5] Collee J. G., Marr W., Collee G. J., Fraser G. A., Marmon B. P., Simmons A. (2008). Specimen collection, culture containers and media. *Mackie and McCartney Practical Medical Microbiology*.

[6] Collee J. G., Miles R. S., Watt B., Collee G., Fraser A. G., Marmon B. P., Simmons A. (2008). Tests for identification of bacteria. *Mackie and McCartney Practical Medical Microbiology*.

[7] Bauer A. W., Kirby W. M., Sherris J. C., Turck M. (1966). Antibiotic susceptibility testing by a standardized single disk method. *American Journal of Clinical Pathology*.

[8] Clinical and Laboratory Standards Institute (2012). *Performance standards for antimicrobial susceptibility testing; 22^nd^ informational supplement, CLSI document M100-S22*.

[9] Banerjee M., Chaudhary B. L., Shukla S. (2015). Prevalence of ESBL and MBL in Acinetobacterspecies isolated from clinical samples in a tertiary care hospital. *Proceedings of the International Journal of Science and Research*.

[10] Sadhana C., Smita W., Charan D., Khare A. S. (2014). Antibiotic resistance pattern of Pseudomonas aeruginosawith special reference to Imipenem and metallo-beta lactamase production. *Indian Journal of Basic and Applied Medical Research*.

[11] Senthamarai S., Sivasankari S., Kumudhavathi M. S. (2013). Susceptiblity pattern of ESBL Strains of P. aeruginosa in a Tertiary Care Hospital, Kanchipuram, Tamilnadu. *International Journal of Recent Scientific Research*.

[12] Goel V., Hogade S., Karadesai S. (2013). Prevalence of extended-spectrum beta-lactamases, AmpC beta-lactamase, and metallo-beta-lactamase producing Pseudomonas aeruginosa and Acinetobacter baumannii in an intensive care unit in a tertiary care hospital. *Journal of the Scientific Society*.

[13] Rani S. V., Rao K. R., Ravinder S., Kanakadurga P. (2016). Prevalence of extended spectrum beta lactamases (ESBL) producing Pseudomonas aeruginosa isolates from burn patients. *Proceedings of the International Journal of Contemporary Medical Research*.

[14] Woodford N., Zhang J., Kaufmann M. E. (2008). Detection of *Pseudomonas aeruginosa* isolates producing VEB-type extended-spectrum *β*-lactamases in the United Kingdom. *Journal of Antimicrobial Chemotherapy*.

[15] Lim K. T., Yasin R. M., Yeo C. C. (2009). Genetic fingerprinting and antimicrobial susceptibility profiles of *pseudomonas aeroginosa* hospital isolates in Malaysia. *Journal of Microbiology and Immunology*.

[16] Upadhyay S., Sen M. R., Bhattacharjee A. (2010). Presence of different beta-lactamase classes among clinical isolates of *Pseudomonas aeruginosa* expressing AmpC beta-lactamase enzyme. *The Journal of Infection in Developing Countries*.

[17] Sharma M., Sarita S., Chaudhary U. (2010). Metallo-beta-lactamase producing Pseudomonas aeruginosa in neonatal septicemia. *Journal of Laboratory Physicians*.

[18] Behera B., Mathur P., Das A., Kapil A., Sharma V. (2008). An evaluation of four different phenotypic techniques for detection of metallo-*β*-lactamase producing *Pseudomonas aeruginosa*. *Indian Journal of Medical Microbiology*.

[19] Aggarwal S., Durlabhji P., Gupta S. (2017). Incidence of Metallo-*β*-lactamase producing *Pseudomonas aeruginosa* isolates and their antimicrobial susceptibility pattern in clinical samples from a tertiary care hospital. *International Journal of Research and Review*.

[20] Sinha M., Srinivasa H., Macaden R. (2007). Antibiotic resistance profile & extended spectrum beta-lactamase (ESBL) production in Acinetobacter species. *Indian Journal of Medical Research*.

[21] Vahaboglu H., Coskunkan F., Tansel O. (2001). Clinical importance of extended-spectrum *β*-lactamase (PER-1-type)-producing *Acinetobacter spp*. and *Pseudomonas aeruginosa* strains. *Journal of Medical Microbiology*.

[22] Chaudhary M., Payasi A. (2012). Molecular characterization and antimicrobial susceptibility study of Acinetobacter baumannii clinical isolates from middle east, African and Indian patients. *Journal of Proteomics & Bioinformatics*.

[23] Bali E. B., Açik L., Sultan N. (2010). Phenotypic and molecular characterization of SHV, TEM, CTX-M and extended-spectrum *β*-lactamase produced by *Escherichia* coli, *Acinobacter baumannii* and *Klebsiella* isolates in a Turkish hospital. *African Journal of Microbiology Research*.

[24] Irfan S., Zafar A., Guhar D., Ahsan T., Hasan R. (2008). Metallo-*β*-lactamase-producing clinical isolates of *Acinetobacter* species and *Pseudomonas aeruginosa* from intensive care unit patients of a tertiary care hospital. *Indian Journal of Medical Microbiology*.

[25] Kabbaj H., Seffar M., Belefquih B., ETAL (2013). Prevalence of Metallo-*β*-Lactamases Producing *Acinetobacter baumannii* in a Moroccan Hospital. *ISRN Infectious Diseases*.

[26] Gupta V., Datta P., Chander J. (2006). Prevalence of metallo-*β* lactamase (MBL) producing *Pseudomonas* spp. and *Acinetobacter* spp. in a tertiary care hospital in India. *Infection*.

